# Pharmacovigilance of cutaneous adverse drug reactions in associations with drugs and medical conditions: a retrospective study of hospitalized patients

**DOI:** 10.1186/s40360-022-00603-4

**Published:** 2022-08-10

**Authors:** Lei Zheng, Hao-bin Jin, Yu-yao Guan, Jing Yang

**Affiliations:** 1 Department of Pharmacy, Shandong Provincial Third Hospital, Cheeloo College of Medicine, Shandong University, Jinan, Shandong P.R. China; 2 School of Medicine and Pharmacy, Ocean University of China, 5 Yushan Road, Qingdao, Shandong P.R. China; 3 Department of Pharmacy, Shandong Medical College, 5460 Erhuannan Road, Jinan, Shandong P.R. China

**Keywords:** Cutaneous reactions, Adverse drug reaction, Abnormal liver and kidney function, Case-non-case study

## Abstract

**Background:**

Cutaneous adverse drug reaction (CADR) is a common problem in clinical medication. This study aimed to investigate the correlation between clinical drug application and CADR occurrence as evidence for preventive strategies and rational clinical drug use.

**Methods:**

We analyzed the characteristics of CADRs of 858 patients admitted to Shandong Provincial Third Hospital from March 2007 to December 2018. The most significant drugs concerning the common skin symptoms and their significance to CADR were investigated by case-non-case and multiple logistic regression analyses.

**Results:**

A total of 266 drugs were involved in 858 cases of CADR. Among the ten most relevant medications, primarily antibiotics and herbal injections, and nutritional support drugs, potassium sodium dehydroandrographolide succinate injection, and cefoperazone sodium and sulbactam sodium injection were found to be 2.1 and 1.45 times statistically more prone to CADRs than to other adverse drug reactions (ADRs), respectively. The main route of administration was intravenous (63.16%), with oral administration accounting for 25.19%. There were 747 cases of ADR, 71 of severe ADR, 2 of new and severe ADRs, and 38 cases of new ADR. Overall, 100 cases of CADR exhibited abnormal alanine aminotransferase, aspartate aminotransferase, and serum creatinine levels. The predictive factors for severe CADR occurrence included allergy and smoking histories, cefoperazone sodium, sulbactam sodium injection, levofloxacin lactate and sodium chloride injection.

**Conclusions:**

Drug-induced CADR symptoms are commonly associated with other ARDs, predominantly rashes and pruritus, and are often accompanied by some medical conditions, especially liver and kidney damage. Detailed attention to a patient’s primary diseases, allergy history, and drug safety profile could help prevent or reverse CADR in most patients.

## Background

Adverse drug reactions (ADRs) are harmful reactions to drugs under normal usage conditions. Approximately 1/3–1/4 of ADRs are skin-related; thus, the term cutaneous ADR (CADR) emerged. CADR has complex pathogenesis involving a variety of clinical manifestations and challenging diagnoses, with the most common symptoms being drug-induced dermatitis or drug rash [1]. Clinicians should promptly deal with CADRs to reduce their harmful effects and use drugs rationally. This study aimed to investigate the correlation between clinical drug administration and CADR occurrence to support clinical prevention strategies and rational drug prescription. Here, we collected 1973 ADRs reported from July 2007 to December 2018 in Shandong Provincial Third Hospital and analyzed 858 reported CADRs to identify their distribution patterns and investigate the influence of some medications on CADR.

## Methods

### Case data

All patients’ details were obtained from the National Adverse Drug Reaction Monitoring System, freely available to hospitals in China. Hospitals report observed adverse drug reactions through the system in a unified format. Researchers or clinicians can search and analyze the reported adverse drug reactions by the name of the drug, the name of the adverse reaction, and the severity.

A total of 1973 patients with ADRs from July 2007 to December 2018 were assessed, of which 858 (43.5%) were suspected to be CADRs. The patients' gender, age, clinical manifestations, liver and kidney function parameters, CADR type, and the drugs involved were analyzed, focusing on severe CADR cases.

### Determination criteria

ADRs were determined according to the “Administrative Measures for Reporting and Monitoring of Adverse Drug Reactions” implemented on March 4, 2004, revised and implemented on July 1, 2011 [2]. Severe ADRs were also determined per the “Technical Specifications and Evaluation Criteria for Common Serious Adverse Drug Reactions” issued by the National Center for ADR Monitoring [3]. The names of adverse drug reactions refer to the WHO adverse reaction terminology. The relationships between ADRs and allergenic drugs were categorized as definitely, probably, possibly, or unlikely related to be judged or unable to judge based on the following criteria: 1) reasonable time of administration, allergic-like reactions occurring during or within hours or days after administration; 2) the types of suspected drugs, drugs known to cause allergies or allergy-like reactions based on information from instruction pamphlets, previous studies, or databases; 3) after drug discontinuation or dose reduction, the reaction is relieved or disappears after anti-allergy treatment; 4) re-administration arouses allergic reactions and even anaphylactic shock; and 5) allergy-like reactions that cannot be explained by the related effects of concomitant drug administration, the patient’s disease progression, or other treatments. The classification was defined as: definitely: 1, 2, 3, 4, 5; probably: 1, 2, 3, 5; possibly: 1, 2, 3; unlikely related: not meeting 1, 2, 3, 4, 5; to be judged: the statement is incompletely filled in, awaiting supplementation, or the causal relationship is difficult to identify or lacking supporting studies; and unable to judge: too many items are missing from the statement, the causal relationship is difficult to determine, and the data cannot be supplemented (Table 1). If multiple ACDR symptoms appeared, only the most significant one was included in the study symptom catalog. All medications involved in this study were prescribed and supplied by hospital clinicians and pharmacists, respectively. No multiple medications (among the ten drugs) were prescribed or supplied to the patients during the study.

**Table 1:** ADR evaluation standard

Grade	Meet the criteria
definitely	1, 2, 3, 4, 5;
probably	1, 2, 3, 5;
possibly	1, 2, 3;
unlikely related	not meeting 1, 2, 3, 4, 5;
to be judged	The statement is incompletely filled in, awaiting supplementation, or the causal relationship is difficult to identify or lacks supporting studies;
and unable to judge	Too many items are missing from the statement, the causal relationship is difficult to determine, and the data cannot be supplemented;

### Data analysis

Variables such as age, past medical history, primary disease, administration routes, indications, and final outcome were expressed as counts and percentages. The Chi-square test assessed the predictive factors for severe CADRs, by comparing the counts between the two groups. If the actual frequency was less than 1 or 20% of the cells were less than 5, Fisher’s exact probability correction was used to test the level at α = 0.05, using SPSS version 22.0 (IBM Corp). All single factors with statistical significance(*P* < 0.05)were included as covariates in multiple logistic regression (stepwise model) analysis of ACDR predictive factors. Statistical significance was determined at a 95% confidence interval (*P* < 0.05). The case-non-case analysis was performed on the ten most significant medications inducing CADRs. The reporting odds ratio (ROR) driven by the fractions of individual medication-induced CADR cases was reported. The ratio of total induced CARDs and ADRs (non-case) with a lower 95% CI greater than 1 indicated statistical significance [4].

## Results

### CADR typing

Among 858 cases of CADR in Shandong Provincial Third Hospital, 38 (4.4%) had new ADRs, 71 (8.3%) had severe ADRs, 2 (0.2%) had new and severe ADRs, and 747 (87.1%) had common ADRs, indicative of CADRs being associated with other ADRs.

### Gender and age distribution of patients

Among the 858 CADR cases, 441 (51.4%) were male, and 417 (48.6%) were female. In this study, patients aged 41–65 constituted the largest number of CADR cases (38.46%), with an average estimated incidence of 0.39% (Table 2).

**Table 2:** Composition ratio and incidence of cutaneous adverse drug reactions by age group

Age/years	Number of cutaneous adverse drug reaction (CADR) cases	Composition ratio/%	Cases hospitalized with medication during the same period/(number of cases)	Incidence of CADR/%
1–6	124	14.45	25900	0.48
7–17	33	3.85	5401	0.61
18–40	130	15.15	36700	0.35
41–65	330	38.46	79420	0.42
≥ 66	241	28.09	75136	0.32
Total	858	100	222557	0.39

### Types of clinical manifestations of CADR

The main types of skin disorder in the 858 CADR cases were papule, macule, pruritus, erythema, skin flushing, skin burning, and pustules. The top three drug categories that caused skin disorders were antibiotics, herbal injections, and nutritional support drugs. After drug discontinuation and aggressive symptomatic treatment, patients were cured in 85.5% of cases and improved in 14.5% of cases (Table 3), suggesting that clinical interventions could reverse the majority of CADRs.

**Table 3:** Composition ratio of various rash types

Damage type	Cases	Composition ratio/%	Main drugs involved
Papule	300	34.97	Antibiotics (128 cases), herbal injections (53 cases), cardiovascular drugs (26 cases), nutritional support drugs (33 cases), respiratory drugs (13 cases), digestive drugs (10 cases), hepatoprotective drugs (5 cases), hypoglycemic drugs (7 cases), antiviral drugs (2 cases), others (23 cases)
Macule	207	24.13	Antibiotics (113 cases), herbal injections (40 cases), cardiovascular drugs (10 cases), nutritional support drugs (10 cases), respiratory drugs (10 cases), digestive drugs (11 cases), neurological drugs (13 cases)
Pruritus	177	20.63	Antibiotics (77 cases), cardiovascular drugs (27 cases), nutritional support drugs (22 cases), herbal injections (21 cases), respiratory drugs (5 cases), digestive drugs (6 cases), neurological drugs (5 cases), hepatoprotective drugs (4 cases), lipid-lowering drugs (2 cases), hypoglycemic drugs (1 case), others (7 cases)
Erythema	63	7.34	Antibiotics (25 cases), cardiovascular drugs (7 cases), nutritional support drugs (10 cases), herbal injections (10 cases), respiratory drugs (2 cases), neurological drugs (2 cases), hepatoprotective drugs (3 cases), hypoglycemic drugs (2 cases), others (2 cases)
Skin flushing	55	6.41	Antibiotics (15 cases), cardiovascular drugs (13 cases), nutritional support drugs (7 cases), herbal injections (9 cases), respiratory drugs (5 cases), digestive drugs (2 cases), neurological drugs (2 cases), others (2 cases)
Skin burning	42	4.90	Antibiotics (14 cases), nutritional support drugs (10 cases), hepatoprotective drugs (2 cases), herbal injections (9 cases), respiratory drugs (7 cases)
Pustules	14	1.63	Antibiotics (10 cases), herbal injection (3 cases), nutritional support drugs (1 case)
Total	858	100.00	

### Drugs involved in CADR

Of the 858 cases of ADR reports recording a suspected CADR, 266 drugs were involved, including 234 synthetic (87.97%) and 32 herbal drugs (12.03%). Overall, 168 of these drugs were administered as injections (63.16%), 67 as oral preparations (25.19%), and 31 as topical applications (11.65%). Of the 266 drugs, the top 10 caused 240 cases of CADR (27.97% of all CADR cases) (Table 4), all administered as injections. Seven were antibacterial drugs, causing 181 cases (21.10%). Among the ten medications, potassium sodium dehydroandrographolide succinate injection and cefoperazone sodium and sulbactam sodium injection were 2.1 and 1.45 times statistically more prone to CADRs than other adverse drug reactions (ADRs) (Table 1). The distributions of the age, past medical history, and primary disease factors in CADR were analyzed and demonstrated that patients over 66 years of age (46.48%) with smoking history (16.09%) and cardiovascular diseases (40.85%) were more prone to CADR than their respective groups (Table 1).

**Table 4:** Top 10 drugs for cutaneous adverse drug reactions

Medication	ACDR Cases	Composition ratio (%)^a^	Total medication supplied^b^	Total ADR Cases (Non-CASE)	Frequency^c^	OR^d^	ROR (95%CI)
**Antibiotics**							
** Cefoperazone sodium and sulbactam sodium injection**	45	5.24	7117	71	6.3	1.457	1.45 (95% CI: 1.08–1.84)*
** Ceftriaxone sodium injection**	30	3.50	1445	42	20.8	1.643	1.64 (95% CI: 0.68–2.61)
** Levofloxacin mesylate and sodium chloride injection**	27	3.14	1585	46	17.0	1.350	1.35 (95% CI: 0.87–1.83)
** Levofloxacin mesylate injection**	26	3.03	1549	49	16.8	1.220	1.22 (95% CI: 0.74–1.70)
** Piperacillin sodium and tazobactam sodium injection**	21	2.45	4507	32	4.7	1.509	1.50 (95% CI: 0.95–2.07)
** Potassium sodium dehydroandrographolide succinate injection**	21	2.45	7309	23	2.9	2.10	2.10 (95% CI: 1.50–2.70)*
** Ciprofloxacin lactate injection**	17	1.98	563	30	30.2	1.303	1.30 (95% CI: 0.70–1.90)
** Levofloxacin lactate and sodium chloride injection**	15	1.75	1751	36	8.6	0.95	0.95 (95% CI: 0.35–1.57)
** Herbal medicine**							
** Safflower injection**	15	1.75	799	15	18.8	1.380	1.38 (95% CI: 0.73–2.02)
** Sanqi *Panax notoginseng* injectionLevofloxacin lactate and sodium chloride injection**	23	2.68	5822	35	4.0	1.511	1.51 (95% CI: 0.98–2.04)
** Total**	240	27.97					

^a^% of total CADR cases (858); ^b^total medication prescribed and supplied by the hospital; ^c^ACDR case number per 1000 users; ^d^odds ratio of the case verses the non-case obtained by Case-non-case analysis; *Reporting adds ratio (ROR), the lower bound of the 95% CI greater than 1 showing a statistical significance

**Table 5:**
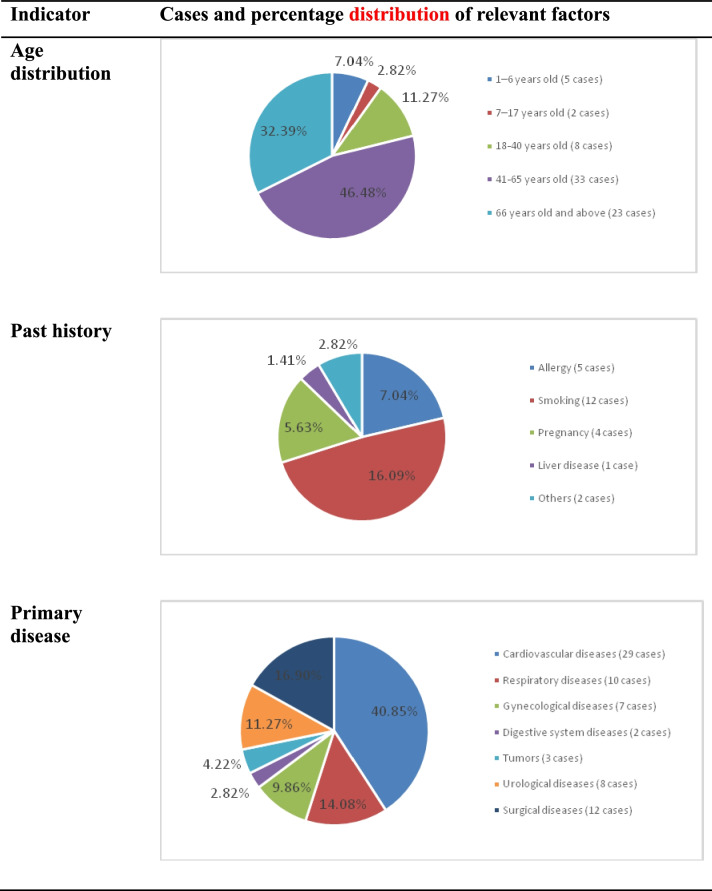
Severe cutaneous adverse drug reactions

### Analysis of severe CADR

There were 71 cases of severe and new severe CADR, with the drugs administered as injections (59 cases, 83.10%) and oral preparations (12 cases, 16.90%). CADRs mainly consisted of severe erythema multiforme drug rash (62 cases, lasting 1–2 days), epidermolysis bullosa drug rash (five cases, lasting 1–4 days), exfoliative dermatitis drug rash (two cases, lasting about one month), and other severe drug rashes (two cases, lasting 1–2 days) (Table 1); 56 cases (78.87%) were over 41 years old. After appropriate treatment, 65 cases were cured, and six improved. The factors related to severe ADRs are shown in Table 5. The main associated factors for severe CADRs in patients included a history of allergy and or smoking or during pregnancy, and medical conditions, including cardiovascular and respiratory diseases. The severe CADR case recovery rate (91.55%) was lower than that of the non-CADR case (99.75%).

**Table 6:** Analysis of the factors involved in severe CARDs

Variable	Number of serious adverse reactions cases (%)	Number of no serious cases (%)	Chi-square/ Fisher
Age			X^2^ = 3.770, *P* = 0.152
Pediatric	7(9.86)	150(19.06)	
Adult	41(57.75)	419(53.24)	
Geriatric	23(32.39)	218(27.70)	
Past history			
History of allergy	5(7.04)	2(0.25)	*P* < 0.0001
History of smoking	12 (16.90)	20(2.54)	*P* < 0.0001
During pregnancy	4 (5.63)	10(1.27)	*P* = 0.023
History of liver disease	1(1.41)	5(0.64)	*P* = 0.405
None	49	750	
Primary disease			
Cardiovascular diseases	29(40.85)	283(35.96)	X^2^ = 3.977, *P* = 0.046
Respiratory diseases	10(14.08%)	161(20.46)	X^2^ = 4.302, *P* = 0.038
Gynecological diseases	7(9.86%)	112(14.23)	X^2^ = 1.042, *P* = 0.307
Digestive system diseases	2(2.82%)	59(7.50)	X^2^ = 2.160, *P* = 0.142
Tumors	3(4.23%)	58(7.37)	X^2^ = 0.975, *P* = 0.323
Urological diseases	8(11.27%)	32(4.07)	*P* = 0.120
Surgical diseases	12(16.90%)	82(10.42)	X^2^ = 2.805, *P* = 0.094
Route of administration			X^2^ = 5.954, *P* = 0.015
Oral	12(16.9)	65(8.26)	
Intravenous	59(83.1)	722(91.74)	
Outcome			*P* < 0.0001
Recovery	65(91.55)	785(99.75)	
Improvement	6(8.45)	2(0.25)	
Drug			
Cefoperazone sodium and sulbactam sodium injection	16	29	X^2^ = , *P* < 0.0001
iodixanol injection	14	132	X^2^ = 0.400, *P* = 0.527
Levofloxacin lactate and sodium chloride injection	4	11	X^2^ = , *P* = 0.029
Piperacillin sodium and tazobactam sodium injection	4	17	X2 = , *P* = 0.088
Sanqi Panax notoginseng injection	3	20	X^2^ = , *P* = 0.429
Ioversol Injection	8	83	X^2^ = 0.036,*P* = 0.850
Levofloxacin mesylate and sodium chloride injection	2	25	X^2^ = , *P* = 1.000
Amiodarone hydrochloride injection	2	45	X2 = , *P* = 0.419
Levofloxacin mesylate injection	4	22	X^2^ = , *P* = 0.263
Ceftriaxone sodium injection	4	26	X^2^ = , *P* = 0.303
Potassium sodium dehydroandrographolide succinate injection	2	19	X^2^ = , *P* = 0.690
Ciprofloxacin lactate injection	2	15	X2 = , *P* = 0.645
Safflower injection	2	13	X2 = , *P* = 0.356

The factors significantly associated with CADR are summarized in Table 5. Notably, a history of allergy and or smoking, ongoing pregnancy, cardiovascular and respiratory diseases, drug administration route, CADR outcome, or taking cefoperazone sodium and sulbactam sodium injection, and levofloxacin lactate and sodium chloride injection were significantly different between severe and non-severe cases of CADR (all *P* < 0.05). Furthermore, a multiple logistic regression analysis was performed to identify independent variables or predictive factors for severe CADR. The resulting data are listed in Table 6. A history of allergy and or smoking, drug administration route, CADR outcome, cefoperazone sodium and sulbactam sodium injection, and levofloxacin lactate and sodium chloride injections were explored as predictive factors for severe CADR, of which patients with previous allergy and smoking, administration of cefoperazone sodium and sulbactam sodium injection, or levofloxacin lactate and sodium chloride injection (OR > 1) were more likely to experience CADR than those without these factors. The drug administration route and CADR outcome(OR < 1) were less likely to predict CADR occurrence, while oral drug administration was more predictive of severe CADR than intravenous drug administration. Patients with improved symptoms had a higher CADR occurrence than those fully recovered.

**Table 7:** Multivariate logistic regression analysis of the predictors of CADR

Covariates	B	S.E	Wald	Freedom	*P*	OR	OR with 95% CI	
							Lower limit	Upper limit
History of allergy	3.743	0.901	17.238	1	0.000	42.206	7.213	246.977
History of smoking	2.158	0.447	23.330	1	0.000	8.654	3.605	20.773
During pregnancy	1.341	0.858	2.446	1	0.118	3.823	0.712	20.528
Cardiovascular diseases	0.112	0.335	0.111	1	0.739	1.118	0.580	2.154
Respiratory diseases	0.197	0.424	0.216	1	0.642	1.218	0.531	2.793
Route of drug administration	-2.314	0.516	20.127	1	0.000	0.099	0.036	0.272
Outcome	-5.182	1.166	19.751	1	0.000	0.006	0.001	0.055
Cefoperazone sodium and sulbactam sodium injection	1.912	0.391	23.912	1	0.000	6.770	3.145	14.570
Levofloxacin lactate and sodium chloride injection	1.680	0.645	6.776	1	0.009	5.367	1.515	19.015
Normal dosage	4.592	1.328	11.948	1	0.001	98.663		

### Abnormal liver and kidney function accompanying CADR

Of the 858 CADR cases, liver and kidney function data were available for 360 patients. Excluding 82 patients suffering from liver and kidney diseases, 278 cases were included in the study. Among them, 100 had abnormal liver and kidney functions with76 having abnormal alanine aminotransferase (ALT) and or aspartate aminotransferase (AST) levels indicative of abnormal liver function, 24 abnormal serum creatinine (Cr) level indicatintive of abnormal kidney function, and 21 both abnormalitie. A severe drug rash was detected in 44 cases, with 33 28 and 24patients having abnormal liver or kidney functions, or both respectively.

In the 100 patients with abnormal liver and/or kidney function, the abnormal values differed between age groups. As liver and kidney function progressively decreases, the age-related functional decline may aggregate drug-induced hepatorenal toxicity accordingly. When using these drugs, liver and kidney function should be tested regularly to promote rational drug use and reduce the risk of adverse reactions.

## Discussion

CADRs are the most common adverse reactions [5]. Sensory changes to the patient’s skin should be monitored after administering drugs, and possible adverse reactions should be addressed immediately.

CADRs are divided into common adverse reactions, new adverse reactions, and severe adverse reactions accordingly, with the common adverse reactions being predominant. Common CADRs include rashes, such as macules, local skin reactions, flushing, pruritus, wheals, allergic purpura, erythematous rash, and acute urticaria. Drugs that cause severe adverse reactions include nimodipine, which causes suspected gangrenous allergic purpura [6], and leflunomide, which causes rashes and terminal cyanosis of the fingers in patients with lupus nephritis [7].

Although most adverse cutaneous drug eruptions are mild and mainly localized macule or urticaria, studies have shown that 2% of severe drug eruptions are life-threatening, necessitating special attention to clinical symptoms such as facial edema, overt eosinophilia, mucosal or conjunctival lesions, eye or skin pain, pale skin lesions, and peeling [8]. Severe cutaneous adverse drug reactions (SCARs) include Steven-Johnson syndrome (SJS), toxic epidermal necrolysis, toxic epidermal necrolysis, acute generalized exanthematous pustulosis (AGEP), drug hypersensitivity syndrome or drug reaction with eosinophilia and systemic symptoms (DRESS). Due to the large area of skin lesions in patients with SCARs leading to rapid water loss, large amounts of protein loss, and uncomplicated secondary skin infection, attention should be paid to the water and electrolyte balance and skin lesion care, and prevention of secondary infection during treatment. Particular attention should be paid to strengthening nursing care, such as eye, oral, nasal, and vulva care to avoid infection and local adhesion. In addition to skin damage, clinical patients should pay close attention to relevant tests and examination results to prevent and treat complications because SCARs can involve various visceral systems. After years of exploration and research, the treatment methods for severe drug eruptions are constantly updated. The traditional glucocorticoid and intravenous immunoglobulin therapies are the first-line treatments for SCARs. However, the dose and timing of glucocorticoids remain to be agreed upon, in addition to paying attention to treating and preventing severe drug eruptions. Genetic testing has played an essential role in preventing severe drug eruptions caused by certain drugs. In short, with increasing clinical attention to and exploring the treatment of severe drug eruptions, patients with SCARs are receiving better treatment and management with a greater chance of recovery [9].

In the 858 cases of CADRs, 71 (8.27%) showed severe adverse reactions, and the main administration form was injection (59 cases; 83.10%). Patients over 41 years old made up the largest portion of CADR cases. Primary diseases were predominantly cardiovascular, cerebrovascular, respiratory, surgical, and urological. Past histories included allergies, smoking, alcohol consumption, and intravenous route of administration, which are predictive factors for serious ADRs. After treating severe ADRs, 65 cases (91.55%) were cured, and six (8.45%) improved. The mechanisms of CADRs are complicated, besides drug-induced hypersensitivity reactions and individual influences from the body, genetic factors, primary disease, and medical history.

Synthetic drugs and traditional Chinese medicines accounted for the majority of CADRs. Adverse reactions caused by antibacterial drugs with high dosages or abuse have previously ranked first for all drug types. Therefore, clinicians should use antibacterial drugs in strict accordance with the “Guidelines for Clinical Application of Antibacterial Drugs” [10], and drug monitoring should be improved to reduce the occurrence of CADRS. Herbal injections should be used cautiously, and drug-drug interactions and dosing intervals should be noted [11].

The liver and kidneys are the largest metabolic organs of the body involving drug metabolism [12–15]. The compounding ingredient of hydroxyethyl starch in non-steroidal anti-inflammatory drugs(NSAIDs)increases hepatic and renal toxicity in elderly patients [16]. The pathogenesis of drug-induced liver injury is mainly through interfering cytochrome CYP450 enzyme metabolism to produce hepatotoxicity, including mitochondrial dysfunction and apoptosis. Moreover, the risk factors for drug-induced liver injury are also associated with the genetic polymorphism of gene CYP. Consequently, the genetic polymorphism of gene CYP detection may also be helpful for ADR prevention prior to the prescription of the relevant medications [17].

In the study of drug-induced liver injury, the significance of relevant biomarkers in liver injury, such as cytoprotein-18, macrophage clustering factor receptor, and bone bridge protein, have been verified. Therefore, the changes in the relevant biological markers are monitored, which is of great significance for preventing liver toxicity caused by drugs [18]. A drug-induced kidney injury is often accumulative and dose-dependence. Carrying on with in-depth studies, some potential markers for diagnosing renal cell damage continue to be discovered, such as kidney injury molecules-1 (KIM-1), neutrophil, neutral enzyme-associated lipoxin (NGAL), clusterin and tubular enzyme activity. Therefore, the markers for early prediction and detection of kidney damage may efficiently predict and prevent nephrotoxicity [19]. Generally, the drug contains active compounding ingredients that may have liver toxicity which has not been thoroughly investigated and are critical to improving clinicians’ awareness of potentially hazardous drugs [20].

Among 858 CADR cases, 100 (11.66%) showed abnormal liver and kidney function. The proportion varies among the age groups, with patients over 41 years constituting the highest proportion. This finding indicates that liver and kidney function progressively decreases with age, which may adversely affect the hepatorenal toxicity of drugs. As such, liver and kidney function tests should be performed regularly in patients taking these drugs, thereby improving the rational use of drugs and minimization of adverse reactions. Furthermore, the results from our study may suggest that patients with previous allergies and smoking should be closely monitored following administration of these drugs, including cefoperazone sodium and sulbactam sodium injection, levofloxacin lactate and sodium chloride injection. If CADR occurs, those medications should be withdrawn immediately to avoid deterioration of the condition(s). Because smoking is a risk factor for CADR, cessation of cigarette smoking is highly recommended to reduce the risk of developing CADR and ensure an effective treatment(s) for the relevant medical condition(s). Skin tests may prevent systematic severe allergy for patients with a history of allergy.

It may merit the attention that several independent risk factors for severe CADR were identified in this study, mainly including a history of allergy, previous smoking, cefoperazone sodium, sulbactam sodium injection, levofloxacin lactate and sodium chloride injection. Our findings support the complex interplay between drugs and host factors. The risk of drug allergy is assessed by consideration of multiple factors [21–24]: 1) Drug factors: nature of the drug, dose, frequency, and duration of drug exposure, route of administration, and cross-sensitization; 2) Host factors: age, gender, genetic predisposition (e.g., human leukocyte antigen type and acetylation status), concomitant diseases (e.g., Epstein-Barr virus, human immunodeficiency virus, and asthma), past drug reactions, and multiple allergic syndromes. Despite lower incidence relative to non-severe CADRs, severe CADRs are potentially life-threatening during the acute stage and associated with severe chronic sequelae, mortality, and high healthcare costs. It has been recognized that early prediction and diagnosis are critically crucial for the timely implementation of appropriate interventions or even urgent drug withdrawal to prevent severe CADRs and long-term sequelae [25]. As such, the identified independent risk factors for severe CADRs have a potential predictive value in creating preventive strategies, navigating medical decisions, and improving patient care. However, we realized the limitations of this study, mainly owing to its retrospective nature. For instance, this study retrospectively analyzed data in a spontaneous reporting system, and no causality was ascribed to any medication used. Therefore, prospective investigations are required to verify the findings of this study.

## Conclusions

CADRs are commonly associated with other ADRs and medical conditions, especially abnormal liver and kidney function. Drug-induced CADR symptoms are predominantly rashes and pruritus and are preventable or reversible by clinical attention to drug safety in CADR. Clinicians should develop individualized dosage regimens for rational drug selection, focus on CADR monitoring in elderly patients and patients with histories of allergy and smoking, given cefoperazone sodium and sulbactam sodium injection or levofloxacin lactate and sodium chloride injection, and closely observe skin changes in patients after drug administration to address adverse reactions quickly and reduce the occurrence of severe adverse reactions.

## Data Availability

The data that support the findings of this study are available from the corresponding author upon reasonable request.
